# Electrospinning Novel Sodium Alginate/MXene Nanofiber Membranes for Effective Adsorption of Methylene Blue

**DOI:** 10.3390/polym15092110

**Published:** 2023-04-28

**Authors:** Meng Li, Pingxiu Zhang, Qianfang Wang, Ningya Yu, Xiaomin Zhang, Shengpei Su

**Affiliations:** National and Local Joint Engineering Lab for New Petro-Chemical Materials and Fine Utilization of Resources, Hunan Normal University, Changsha 410081, China; lm@smail.hunnu.edu.cn (M.L.); ZPX_serendipity@163.com (P.Z.); wqf199801@163.com (Q.W.); sushengpei@gmail.com (S.S.)

**Keywords:** sodium alginate, MXene, electrospinning, methylene blue, adsorption

## Abstract

Understanding how to develop highly efficient and robust adsorbents for the removal of organic dyes in wastewater is crucial in the face of the rapid development of industrialization. Herein, d-Ti_3_C_2_T_x_ nanosheets (MXene) were combined with sodium alginate (SA), followed by electrospinning and successive Ca^2+^-mediated crosslinking, giving rise to a series of SA/MXene nanofiber membranes (NMs). The effects of the MXene content of the NMs on the adsorption performance for methylene blue (MB) were investigated systemically. Under the optimum MXene content of 0.74 wt.%, SA/MXene NMs possessed an MB adsorption capacity of 440 mg/g, which is much higher than SA/MXene beads with the same MXene content, pristine MXene, or electrospinning SA NMs. Furthermore, the optimum SA/MXene NMs showed excellent reusability. After the adsorbent was reused ten times, both the MB adsorption capacity and removal rate could remain at 95% of the levels found in the fresh samples, which indicates that the electrospinning technique has great potential for developing biomass-based adsorbents with high efficiency.

## 1. Introduction

The pollution of water bodies by dyes has attracted increasing attention due to the continuous expansion of the relative application areas, including the textile, paper, printing, pulp mill, food, and plastics industries, etc. [1,2,3,4]. Methylene blue (MB) is a typical cationic dye with an aromatic heterocycle and has been the most commonly used for dyeing cotton, wool, and silk [5,6]. Although MB is not considered to be highly toxic, acute exposure to MB would cause eye burns in humans and animals, and if ingested, nausea, vomiting, diarrhea, and quadriplegia [7]. Indeed, MB has been studied as one of the typical model compounds for removing organic contaminants and colored bodies from aqueous solutions, due to its strong adsorbability onto solids, nonbiodegradability, and widespread use in the industry [8,9]. With the rapid development of industrialization, the discharge of dye-containing wastewater is leading to increased environmental concerns. In this context, it is desirable to find efficient methodologies for the removal of dyes from wastewater prior to its disposal in the natural environment.

So far, several methodologies have been developed for the treatment of dye-containing wastewater, including membrane separation [10], biodegradation [11], flocculation [12], chemical oxidation [13], photocatalytic degradation [14], and adsorption [15]. Of the above processes, adsorption has been deemed as one of the most promising methods due to its environment friendliness, such as low cost/energy consumption, high selectivity/efficiency, and simplicity of operation [16]. Recently, a variety of adsorbents associated with two-dimensional (2D) materials, for example, graphene [17], molybdenum disulfide [18], and MXene [19], have shown great potential in the adsorption of dyes. These 2D materials possess high specific surface areas and numerous/tunable terminal groups, which are, theoretically, conducive to the adsorption of foreign species [20,21]. MXenes, a group of novel 2D materials composed of transition metal carbides and/or nitrides, have some unique properties, including highly active layered structures with hydrophilic terminal groups (-F, -OH, and -O- groups, etc.) and excellent dispersity in aqueous solution originating from the abovementioned hydrophilic groups [22,23,24], which make such materials particularly suitable for the treatment of wastewater.

It should be noted that, although some adsorbents based on MXenes showed good performance for the removal of MB in wastewater [25,26], the fast deterioration of adsorption capacity originating from self-aggregation/self-restacking, because of the strong van der Waals force interaction between adjacent nanosheets, together with the difficulty of solid–liquid separation in the aqueous solution due to good hydrophilicity, have seriously hindered the application of pristine MXenes in the treatment of wastewater [27,28]. To overcome the above drawbacks, several studies have explored the incorporation of foreign species with MXenes to elevate physicochemical stability and reusability during the adsorption processes [29,30,31,32]. Ti_3_C_2_ MXene nanosheets have been functionalized by magnetic Fe_3_O_4_ nanoparticles and the resultant MXene@Fe_3_O_4_ composites tackle the separation problem via magnetic collection in the adsorption of MB, although the adsorption capacity is relatively low [33]. In contrast, the incorporation of some organic polymers, for example, polyacrylamide [34] and polydopamine [35], with MXenes leads to significantly increased adsorption capacity, separation efficiency, and treating throughput during the adsorption of MB. In this context, organic polymers with suitable structure/functional moieties seem well positioned to address the challenges of developing efficient MXene-containing adsorbents with excellent physicochemical stability and reusability.

More recently, owing to their biodegradability, non-toxicity, and affordability, biomass-based polymers have been reported to have been incorporated with MXenes for the treatment of wastewater [36,37]. Sodium alginate (SA), being a natural linear polysaccharide copolymer derived from seaweed, has shown a good performance in adsorbing MB, due to the fact that its electronegative nature leads to an electrostatic interaction with the electropositive MB [38,39]. Furthermore, the abundant -OH and -COOH groups presented in SA make this natural polymer easily crosslinkable via the ion-exchange with various multivalent cations, leading to good physicochemical stability of the ultimate materials [40,41,42]. MXene/SA beads [28] and MXene/poly-ethylenimine/SA aerogels [43] have been fabricated via Ca^2+^-mediated crosslinking processes for the removal of MB and Cr (VI) species, respectively. It should be noted that, although multivalent cation-mediated crosslinking endows the composite adsorbents with physicochemical stability, MXene nanosheets could be buried and/or poorly dispersed in the SA networks, which would lower the adsorption performance of the composite adsorbents.

In a continuation of our efforts to explore the applications of biomass-based materials in wastewater treatment [44], herein a series of SA/MXene nanofiber membranes (NMs) were prepared via electrospinning and successive Ca^2+^-mediated crosslinking processes, which has been demonstrated to be an efficient technique for fabricating continuous nanoscale fibers with diameters in the range of tens of nanometers to sub-micrometers [45,46,47,48]. The resultant SA/MXene NMs exhibited a superior MB adsorption capacity to that of SA/MXene beads, pristine MXene, or electrospinning SA NMs, because the three-dimensional fibrous nanostructure makes it easy for the MB to access the adsorption sites of the SA/MXene composite NMs. To the best of our knowledge, despite the fact that there have been a few prior studies on fabricating electrospinning polymer-based NMs for the adsorption of MB [49,50,51], this is the first example associated with the fabrication of electrospinning SA/MXene NMs and the relevant adsorption application for the treatment of dye-containing wastewater.

## 2. Materials and Methods

### 2.1. Materials

Ti_3_AlC_2_ powder (400 mesh) was purchased from 11 Technology Co., Ltd. (Changchun, China). Sodium alginate (SA, 20 mPa·s, CP), chitosan (CS, 100–200 mPa·s, CP), calcium chloride dihydrate (CaCl_2_·2H_2_O, 99%, AR), hydrochloric acid (HCl, 37%, AR), methylene blue (MB, C_16_H_18_ClN_3_S, AR), and methyl orange (MO, C_14_H_14_N_3_NaO_3_S, AR) were purchased from Sinopharm chemical reagent Co., Ltd. (Shanghai, China). Lithium fluoride (LiF, 99%, AR) was purchased from Rhawn (Shanghai, China). Polyvinyl alcohol (PVA-1788) was purchased from Macklin (Shanghai, China). Deionized water was homemade. All reagents are used directly without further purification.

### 2.2. Preparation of d-Ti_3_C_2_T_x_ (MXene)

MXene was synthesized according to the method reported previously [52]. Briefly, 3.00 g of LiF was added to 60.0 mL of HCl (9.0 M) in a PTFE bottle. After stirring for 0.5 h in an ice bath, 3.00 g of Ti_3_AlC_2_ was slowly added and the resultant mixture was stirred at 35 C for 24 h. The obtained multilayer Ti_3_C_2_T_x_ was washed with deionized water and centrifuged at 3500 rpm for 5 min. The above process was repeated until the pH value of the supernate was close to 7.0. Deionized water was added to the obtained multilayer Ti_3_C_2_T_x_ and the mixture was sonicated for 1 h under nitrogen protection. Finally, a delaminated Ti_3_C_2_T_x_ (d-Ti_3_C_2_T_x_, MXene) colloidal solution (ca. 1.5 mg/mL) was obtained by centrifuging the sonicated mixture at 3500 rpm for 60 min to eliminate the sediment.

### 2.3. Preparation of Electrospinnable SA/MXene Solution

A homogeneous and electrospinnable SA/MXene solution was obtained by blending aqueous SA solution (2 wt.%), PVA solution (10 wt.%), and the above MXene solution (ca. 1.5 mg/mL). More specifically, the SA solution was prepared by dissolving 2.00 g of SA in 98.00 g of deionized water at room temperature under continuous stirring; the PVA solution, as a spinning auxiliary, was prepared by adding 10.00 g of PVA to 90.00 g of deionized water under continuous stirring at room temperature for 1 h and, subsequently, at 90 °C for 6 h. Finally, the electrospinnable solution was obtained by mixing SA, PVA, and the desired amount of MXene solution under stirring at room temperature for 12 h, wherein the volume ratio of the PVA solution to the SA solution was 7:3.

### 2.4. Fabrication of SA/MXene NMs

**Scheme 1:**
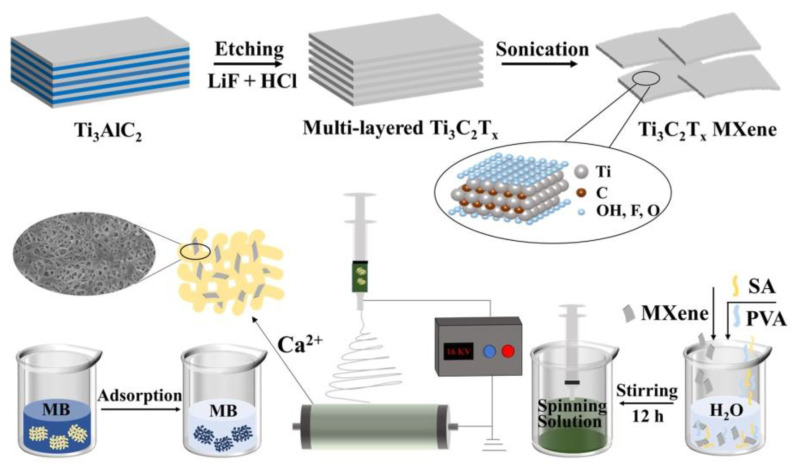
Schematic illustration for the fabrication of SA/MXene NMs.

As illustrated in Scheme 1, the individual electrospinnable SA/MXene solution with various MXene contents was loaded into a 10 mL syringe capped with a 23 gauge stainless steel needle. The solution was fed through the needle at a rate of 0.1 mm/min, the working distance between the needle tip and the collector was set to 10 cm, and the working voltage was 16 Kv (Ucalery, Yongkang Leye Co., LTD, Beijing, China). Electrospinning was performed at room temperature and 60% relative humidity. The resultant NMs were immersed in 50.0 mL of CaCl_2_ solution (1.0 M) for 12 h at room temperature, followed by washing with plenty of deionized water several times; the resultant solid was then submerged in plenty of deionized water without agitation for 5 h at room temperature to remove residual PVA. The soaked SA/MXene NMs were dried under vacuum at 60 C and then sealed for the subsequent adsorption experiments. The designations of the SA/MXene NMs were made according to the content of MXene in the sample. For example, the sample denoted SA/MX-0.74 NMs had an MXene content of 0.74 wt.%. For comparison purposes, SA NMs without MXene were prepared according to the above processes. In addition, SA beads and SA/MXene beads with 0.74 wt.% of MXene were prepared by dropping the corresponding solutions into CaCl_2_ solution (1.0 M) directly, and were denoted as SA-Bs and SA/MX-Bs, respectively.

### 2.5. Adsorption Measurements

The adsorption abilities of the samples towards MB were systematically investigated using a water bath shaker (150 rpm). Firstly, MB was dissolved in deionized water to obtain 1000 mg/L stock solution. The effects of operational factors, such as contact time (0–48 h), pH value of solution (2.0–11.0), initial MB concentration (50–220 mg/L), adsorbent dosage (1–5 mg), and adsorption temperature (298–323 K) were evaluated to examine the adsorption properties of the samples. The pH value of the MB solution was adjusted by 0.1 M HCl or 0.1 M NaOH. After the adsorption experiments, the MB concentration of the solutions (diluted five times) was measured using UV-vis at room temperature. The adsorption capacity (Q_e_) and removal efficiency (R) were evaluated by using the following expression:

(1) $$Q_{t}=\frac{C_{0}-C_{t}}{m}\;\times \;V,$$

(2) $$Q_{e}=\frac{C_{0}-C_{m}}{m}\;\times \;V,$$

(3) $$R=\frac{C_{0}-C_{e}}{C_{0}}\;\times \;100\%,$$

where C_0_ and C_e_ are the initial and equilibrium concentrations of MB (mg/L), respectively; V and m are the volume of the MB solution (L) and the weight of the adsorbent dosage (mg), respectively; Q_t_ and C_t_ are the adsorption capacity (mg/g) and the concentration of MB (mg/L) at time t, respectively; and Q_e_ and R are the equilibrium adsorption capacity (mg/g) and the removal rate of MB, respectively.

### 2.6. Desorption and Regeneration

After the adsorption experiment, the sample of SA/MXene NMs was separated simply with tweezers, rinsed several times with deionized water to remove the MB on the surface of the sample, and immersed in 5.0 mL of 5% acidic ethanol for 6 h. After being rinsed with deionized water, the samples were subjected to the above elution process again to guarantee the complete removal of the MB. Finally, the sample was washed with plenty of deionized water and dried under vacuum for subsequent use. During reuse, the sample was treated according to the aforementioned procedure after each adsorption.

### 2.7. Characterization

Scanning electron microscopy (SEM) images were observed on a ZEISS Sigma 300 microscope, operated at 3 kV, 20 C. Prior to the SEM observations, all the samples were coated with a thin layer of gold using the Oxford Quorum SC7620 plasma sputtering apparatus. Transmission electron microscopy (TEM) images were observed on a JEOL JEM-2100 microscopy, operated at 200 kV, 20 C. Fourier transformation infrared (FTIR) spectra were obtained on an AV ATAR 370 Thermo Nicolet spectrophotometer from 600 to 4000 cm^−1^ using KBr pellets. The X-ray diffraction (XRD) patterns were recorded on a Rigaku Ultima IV diffractometer using CuKα radiation (λ = 0.154 nm), operated at 40 kV and 40 mA with scan angle from 2θ = 5–80° at a scan rate of 10° min^−1^. Ultraviolet-visible light (UV-vis) spectra were recorded on a Shimadzu UV-2450 spectrophotometer by using a quartz cuvette with an optical path length of 1 cm at room temperature. Thermogravimetric (TGA) curves were obtained on a NETZSCH STA 2500 simultaneous thermal analyzer under nitrogen atmosphere from 25 to 800 C with a heating rate of 10 C min^−1^. The Zeta potentials (ζ) were measured by using a MALVERN ZETASIZER NANO ZS90 instrument at 25 C, the individual potential was measured for three times for accuracy.

## 3. Results

### 3.1. Adsorption Performance of Various Adsorbents

**Figure 1:**
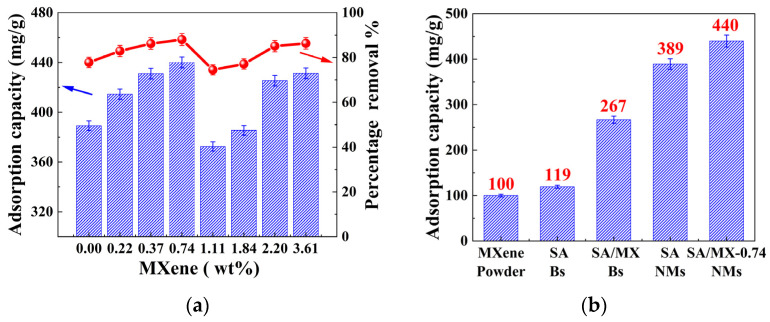
(**a**) MB adsorption performance of SA/MXene NMs with different MXene content. (**b**) MB adsorption performance of various adsorbents. (C_0_ = 50 mg/L, V = 30 mL, T = 298 K, pH = 7.0, dosage = 3 mg, t = 24 h).

Since both d-Ti_3_C_2_T_x_ [26] and Ca^2+^-crosslinked SA [38] have showed good performance in adsorbing MB, the effects of MXene content in SA/MXene NMs on adsorption performance were investigated first. As shown in Figure 1a, when the MXene content increased from 0 to 1.11 wt.%, the adsorption capacity gradually increased and reached maxima of 440 mg/g at an MXene content of 0.74 wt.%, and then dropped to 373 mg/g at an MXene content of 1.11 wt.%. Surprisingly, the samples of SA/MX-1.11 NMs and SA/MX-1.84 NMs showed rather low adsorption capacities for MB, which were even lower than that of the pristine SA NMs (see Figure 1b). The reason for the inferior adsorption capacities observed for the two samples remains unclear. It is possible that both d-Ti_3_C_2_T_x_ and SA are electronegative [53,54], which leads to a non-synergistic effect in the range of certain content levels of MXene. It should be noted that when the content was higher than 1.84 wt.%, the adsorption capacities of the samples exceeded those of the pristine SA NMs (see Figure 1a). Moreover, the effective adsorption capacities of MXene in SA/MX-2.20 NMs and SA/MX-3.61 NMs, calculated by subtracting the adsorption capacity of the pristine SA NMs, were as high as 2045 and 1551 mg/g, respectively, which were much higher than the adsorption capacity of MXene powder (100 mg/g, see Figure 1b). These results suggest that the self-restacking level of MXene is relatively low in the samples of SA/MX NMs, even with the high MXene content presented here.

As expected, owing to the self-restacking, the MXene powder showed a relatively low adsorption capacity of 100 mg/g for MB (Figure 1b), in line with the results reported previously [26]. The sample of SA-Bs possessed a similar adsorption capacity to that of the MXene powder (119 mg/g). It should be noted that the addition of a little MXene powder led to a dramatic increase in the adsorption capacity. The sample of SA/MX-Bs with 0.74 wt.% d-Ti_3_C_2_T_x_ showed an adsorption capacity as high as 267 mg/g, four times higher than that of the reported d-Ti_3_C_2_T_x_/SA beads with 40 wt.% d-Ti_3_C_2_T_x_ [28], which implies that, as for the d-Ti_3_C_2_T_x_/SA composites, a low content of MXene is conducive to the obtainment of high adsorption capacity for MB. In the case of the samples prepared via electrospinning processes, the SA NMs and SA/MX-0.74 NMs possessed much higher adsorption capacities than their spherical counterparts, regardless of whether or not they had MXene content, due to the fact that the three-dimensional fibrous nanostructures originating from the electrospinning made it easy for the MB to access the adsorption sites (vide infra, the photographs of the above adsorbents are shown in Figure S1). Nevertheless, combining the adsorption capacity for MB with the utilization efficiency of MXene, the sample of SA/MX-0.74 NMs with 0.74 wt.% d-Ti_3_C_2_T_x_ was chosen for further research.

### 3.2. Characterization of SA/MX-0.74 NMs

**Figure 2:**
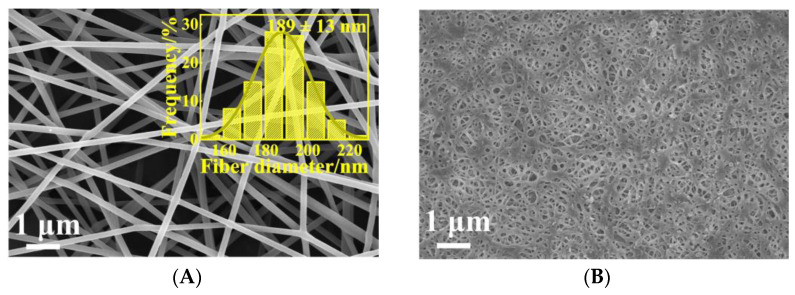

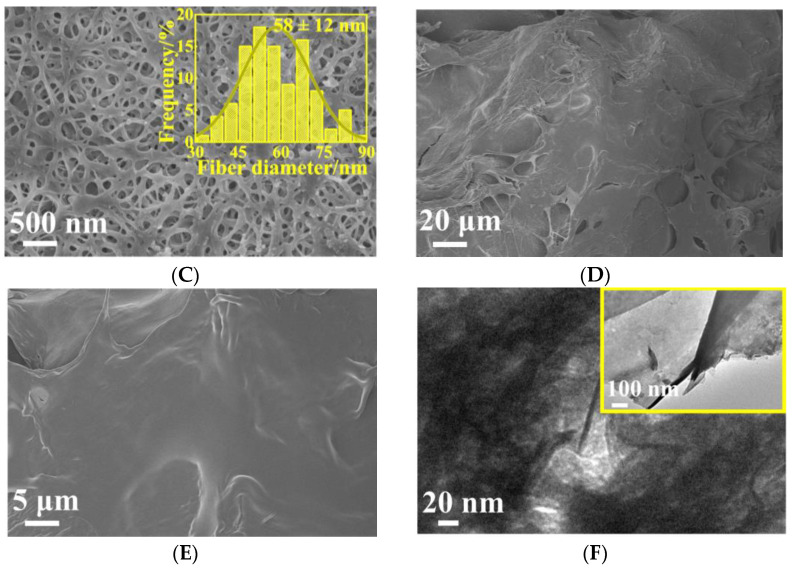
SEM and fiber diameter distribution of (**A**) SA NMs; SEM and fiber diameter distribution of (**B**,**C**) SA/MX-0.74 NMs; SEM of (**D**,**E**) SA/MX-Bs; TEM of (**F**) SA/MX-0.74 NMs and (inset) pristine MXenes.

Owing to the high flexibility of the NM materials presented here, their physicochemical properties could not be measured using a nitrogen adsorption–desorption technique; therefore, SEM observations were invoked to analyze the evolution of their morphology as the MXene was incorporated. The pristine SA NMs showed a similar morphology to the results reported previously [45,48], which comprised large fibers (189 nm) with a few interconnections at the junctions of the fibers (Figure 2a). As shown in Figure 2b, the sample of SA/MX-0.74 NMs with 0.74 wt.% MXene possessed a sponge-like morphology with a thickness of ca. 2 μm (see Figure S2). Careful inspection of the SEM image with high magnification (Figure 2c) indicated that the sponge-like structure consisted of numerous fibers with a smaller diameter of ca. 58 nm (Figure 2c) and more interconnections at the junctions of the fibers as compared with the pristine SA NMs. The XPS spectrum of the SA/MX-0.74 NMs (Figure S3) showed Ti 2p splitting peaks, indicating that it is the existence of MXene that changes the morphology of SA/MX-0.74 NMs. In contrast, the SA/MX-Bs showed a dense structure featuring some irregular micrometer-scale potholes on the surface (Figure 2d,e). TEM observations indicated that the MXene nanosheets (Figure 2f inset) were well-dispersed in the fibers of SA/MX-0.74 NMs (Figure 2f). Combining SEM with TEM observations, it is logical to conclude that the superior adsorption capacity of SA/MX-0.74 NMs, as compared with that of their spherical counterparts the SA/MX-Bs, originates from the unique sponge-like fibrous structure, which affords numerous adsorption sites and allows large MB molecules to penetrate the framework easily.

**Figure 3:**
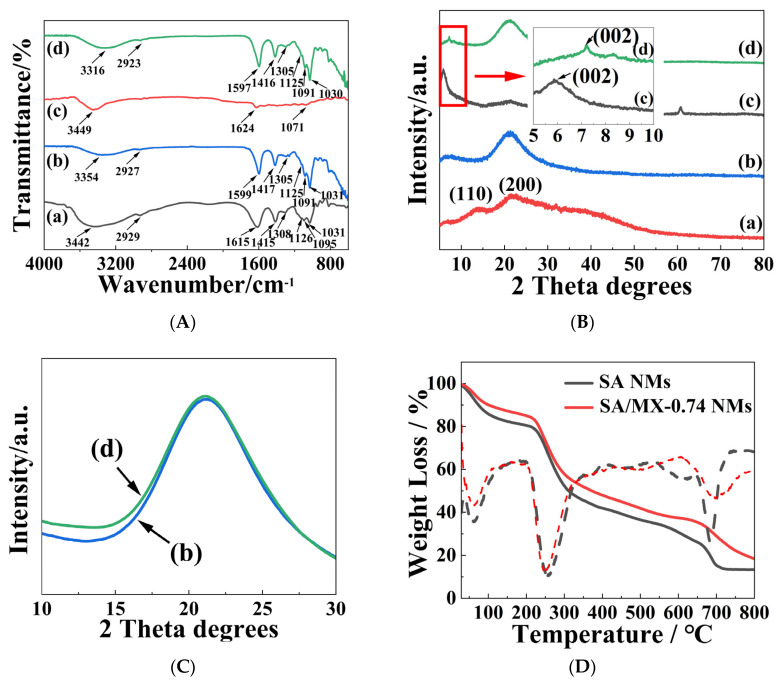
(**A**) FTIR spectra of ((**A**)−a) pristine SA, ((**A**)−b) SA NMs, ((**A**)−c) MXene, and ((**A**)−d) SA/MX-0.74 NMs; (**B**) XRD patterns of ((**B**)−a) pristine SA, ((**B**)−b) SA NMs, ((**B**)−c) MXene, and (d) SA/MX-0.74 NMs; (C) Enlarged XRD patterns of ((**C**)−b) SA NMs and ((**C**)−d) SA/MX-0.74 NMs; (**D**) TGA−DTG curves of SA NMs and SA/MX-0.74 NMs (Solid lines−TGA; Dotted lines−DTG).

Figure 3A displays the FT-IR spectra of the pristine SA, SA NMs, MXene, and SA/MX-0.74 NMs. Both the pristine SA and SA NMs showed vibration bands at 2927–2929, 1415–1417, 1305–1308, 1125–1126, 1091–1095, and 1301 cm^−1^, which can be assigned, respectively, to the C-H stretching vibrational modes of pyranose rings, O-C-O symmetric stretching vibrational modes of carboxylate moieties, C-H deforming vibrational modes of H-C-C and H-C-O moieties in pyranose rings, C-O stretching vibrational modes of hemiacetal moieties in pyranose rings, C-C and C-O stretching vibrational modes of pyranose rings, and C-O stretching vibrational modes of glycosidic bonds [38,55]. After electrospinning and successive Ca^2+^-crosslinked processes, the O-C-O asymmetric stretching vibrational modes of carboxylate moieties at 1615 cm^−1^ shifted to 1599 cm^−1^ and the O-H stretching vibrational modes of hydrogen bonded hydroxyls centered at 3442 cm^−1^ broadened, weakened, and shifted to 3354 cm^−1^ (Figure 3A-a vs. A-b). The former indicated that the crosslinking mediated by Ca^2+^ ions had been accomplished. The latter implied that the hydrogen bond interaction among the hydroxyls of pyranose rings had become stronger, possibly due to the confinement effect originating from the Ca^2+^-mediated crosslinking of the linear polysaccharide structure [56]. The pristine MXene of d-Ti_3_C_2_T_x_ showed O-H stretching vibrational modes of the hydroxyls at 3449 cm^−1^, C-O stretching vibrational modes at 1071 cm^−1^, and C=O stretching vibrational modes at 1624 cm^−1^, similar to the reported results [37,57]. Owing to their relatively low MXene content, the SA/MX-0.74 NMs showed an almost identical spectrum to that of the SA NMs, except that the O-H stretching vibrational modes of hydrogen bonded hydroxyls centered at 3354 cm^−1^ shifted to 3316 cm^−1^. In this context, there existed a strong interaction among the O-containing functional groups of MXene and SA. Indeed, SEM observations had suggested the existence of a strong interaction between the MXene and SA, in which the addition of a little MXene led to smaller fibers with more interconnections at the junctions of these fibers.

The XRD patterns of the pristine SA, SA NMs, MXene, and SA/MX-0.74 NMs are shown in Figure 3B. The pristine SA exhibited two unambiguous diffraction peaks at 13.8°and 21.6°, assigned, respectively, to the (110) plane from the polyguluronate units and the (200) plane from the polymannonate units, and a broad diffraction peak centered at 39°, resulting from the amorphous halo (Figure 3B-a) [58,59]. After electrospinning and successive Ca^2+^-crosslinked processes, the semi-crystalline structure in the pristine SA disappeared and the resultant SA NMs showed a broad diffraction peak centered at 21°, which was due to the fact that the confinement effect resulted from the Ca^2+^-mediated crosslinking destroyed the hydrogen bonding interaction among the linear polysaccharide chains [28]. MXene showed a diffraction peak at 5.8°, which is the characteristic (002) plane of delaminated multilayer Ti_3_C_2_T_x_ [60]. The sample of SA/MX-0.74 NMs doped with MXene exhibited a similar XRD pattern to the SA NMs, aside from the diffraction peak of MXene. Carefully inspecting the patterns of the samples of MXene, SA NMs, and SA/MX-0.74 NMs revealed that, after MXene was combined with SA, the characteristic (002) plane of MXene shifted from 5.8° to 7.2° (Figure 3B inset) and the diffraction peak assigned to amorphous SA became broader (see Figure 3C). Both the decreased interlayer distance of MXene and the broader amorphous diffraction peak of SA indicated that a strong interaction existed between MXene and SA, consistent with the SEM and FT-IR analyses. TGA-DTG curves of the SA NMs and SA/MX-0.74 NMs are shown in Figure 3D. The sample of SA/MX-0.74 NMs showed almost identical weight losses with those of the SA NMs. More specifically, the first weight loss, centered at 65 C, was apparently caused by desorption of physisorbed and chemisorbed water; the second weight loss, centered at 260 C, could be due to the decomposition of pyranose rings; the third weight loss, centered at ca. 700 C, originated from the thermal decomposition of carbon residues [61].

## 4. Adsorption Studies

### The Effects of Operational Factors

**Figure 4:**
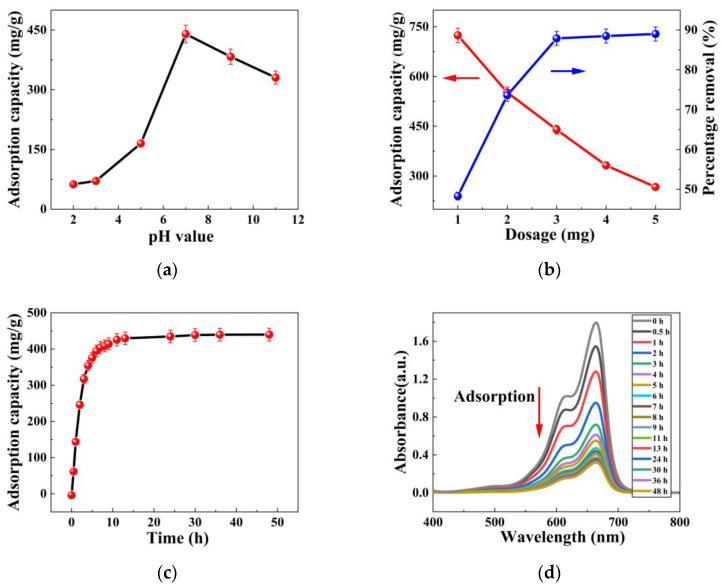

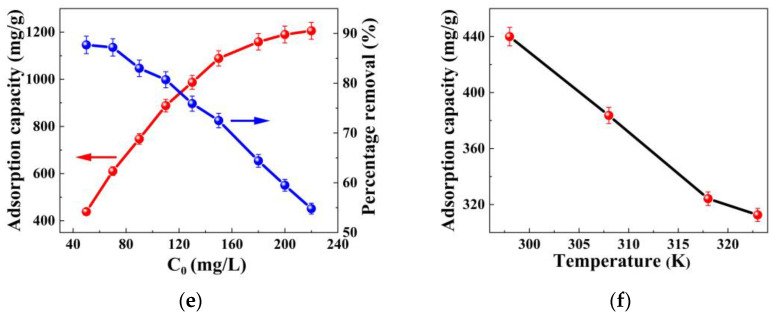
The effects of operational factors on MB adsorption capacity of SA/MX-0.74 NMs. (**a**) pH value: cond. pH = 2.0–11.0, C_0_ = 50 mg/L, V = 30 mL, T = 298 K, t = 24 h, dosage = 3 mg; (**b**) absorbent dosage: cond. dosage = 1–5 mg, C0 = 50 mg/L, V = 30 mL, T = 298 K, t = 24 h, pH = 7.0; (**c**) adsorption time: cond. t = 0–48 h, C_0_ = 50 mg/L, V = 30 mL, T = 298 K, pH = 7.0, dosage = 3 mg; (**d**) time-dependent UV-vis spectra in the adsorption of MB over SA/MX-0.74 NMs; (**e**) initial concentration of MB: cond. C_0_ = 50–220 mg/L, V = 30 mL, T = 298 K, t = 24 h, pH = 7.0, dosage = 3 mg; (**f**) adsorption temperature: cond. T = 298–323 K, V = 30 mL, C_0_ = 50 mg/L, t = 24 h, pH = 7.0, dosage = 3 mg.

Since the pH values in the MB adsorption processes would influence the surface charge and properties of both the adsorbent and adsorbate, the effects of the pH value of the aqueous MB solution on the adsorption capacity of SA/MX-0.74 NMs were investigated first and the results are shown in Figure 4a. Under the situation of relatively low pH values, the sample of SA/MX-0.74 NMs showed inferior adsorption capacities. In these cases, most of the MB existed in molecular form, as the pKa value of MB is equal to 3.8 [62], and was reluctant to adsorb on the moieties of the protonated carboxyls and hydroxyls in the SA/MX-0.74 NMs [63,64]. When the pH value was increased to 5.0, the SA/MX-0.74 NMs showed a moderate adsorption capacity, which should be a compromise result between the ionized carboxyls and the competitive adsorption of protons with MB cations [28,63]. Under neutral condition, the highest adsorption capacity was observed for the sample of SA/MX-0.74 NMs, because the carboxyls of SA were ionized completely, the surface of MXene became electronegative [28], and the competitive adsorption of protons decreased dramatically. Similar to previously reported results [65], the alkaline situation went against the adsorption of MB on the SA/MX-0.74 NMs, which may be due to the Na^+^ cations presented here [66]. The evolution of the zeta potentials of the SA/MX-0.74 NMs as the pH value increased (see Figure S4) further confirmed the above tendency. The SA/MX-0.74 NMs showed zeta potentials close to zero at relatively low pH values and the zeta potential became lower as the pH value increased. Nevertheless, the above observations suggest that neutral condition is suitable for SA/MX-0.74 NMs during the adsorption of MB.

As shown in Figure 4b, when the dosage of SA/MX-0.74 NMs was increased from 1 mg to 5 mg, the adsorption capacity for MB decreased gradually, while the corresponding removal rate increased to ca. 88% at a dosage of 3 mg and then remained unchanged, provided that the dosage exceeded 3 mg. The higher dosage, the greater the number of adsorption sites; therefore, the removal rate initially increased with the increase in the adsorbent dosage. It should be noted that, similarly to previously reported results [65], the SA/MX-0.74 NMs revealed an MB adsorption-limiting concentration of ~6.5 ppm within the dosage presented here. In this context, unchanged removal rates were observed provided that the dosage exceeded 3 mg. Considering the balance between the adsorption capacity and the removal rate, the dosage of 3 mg appears to be suitable for SA/MX-0.74 NMs. Time-dependent adsorption capacity and UV-vis spectra in the adsorption of MB over the SA/MX-0.74 NMs are shown in Figure 4c, d, respectively. The adsorption capacity of the SA/MX-0.74 NMs increased dramatically in the first 5 h, increased slowly in the range 5–24 h, and remained almost unchanged after 24 h. The above adsorption behavior was consistent with that previously reported in MXene/SA beads [28].

As shown in Figure 4e, the adsorption capacity increased with the increase of initial MB concentration, whereas the corresponding removal rate decreased gradually. More specifically, at concentrations lower than 90 mg/mL, the removal rates were higher than 85% and the MB concentrations after the adsorption processes were close to the MB adsorption-limiting concentration of ~6.5 ppm for the adsorbent presented here, which means that the available adsorption sites were sufficient to reach adsorption equilibrium [61,67]. When the initial MB concentration was higher than 70 mg/mL, the limitation of adsorption sites led to a significantly decreased removal rate although the adsorption capacity increased [61,67]. Finally, the effects of adsorption temperature were investigated and the results are shown in Figure 4f. The adsorption capacity of SA/MX-0.74 NMs decreased with the increase in temperature from 298 to 323 K, which indicated that the adsorption processes of MB over the SA/MX-0.74 NMs were exothermic in nature. The unusual relationship between adsorption temperature and adsorption capacity observed for the SA/MX-0.74 NMs possibly originated from the enhanced interaction of MXene and SA framework at high temperature, which would reduce the available adsorption sites [61].

## 5. Adsorption Mechanism

### 5.1. Adsorption Kinetics

The analyses of adsorption kinetics can also permit the inspection of corresponding adsorption mechanisms, which is of great significance in wastewater treatment. In order to verify the adsorption mechanism of SA/MX-0.74 NMs, pseudo-first-order and pseudo-second-order kinetics were invoked to analyze and simulate the adsorption behavior of SA/MX-0.74 NMs, as shown in Equations (4) and (5), respectively [68].

Pseudo-first-order model:

(4) $$\log\left(Q_{e}-{\;Q}_{t}\right){=\mathrm{logQ}}_{e}-{\;K}_{1}t/2.303,$$

Pseudo-second-order model:

(5) $$\frac{t}{Q_{t}}=\frac{1}{K_{2}Q_{e}^{2}}+\frac{t}{Q_{e}},$$

where Q_e_ (mg/g) and Q_t_ (mg/g) are the equilibrium adsorption capacity and the adsorption capacity at time t, respectively; and K_1_ (h^−1^) and K_2_ (g/mg h) are the rate constants of pseudo-first-order and pseudo-second-order adsorption, respectively.

**Figure 5:**
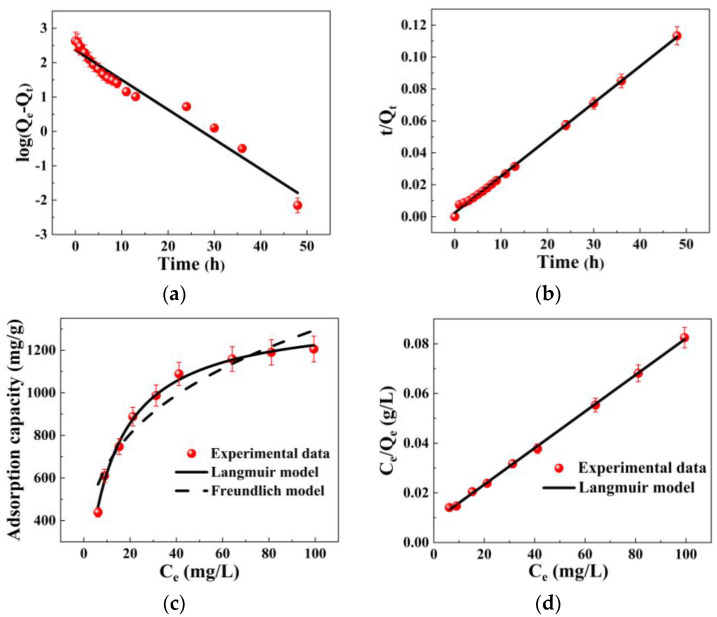

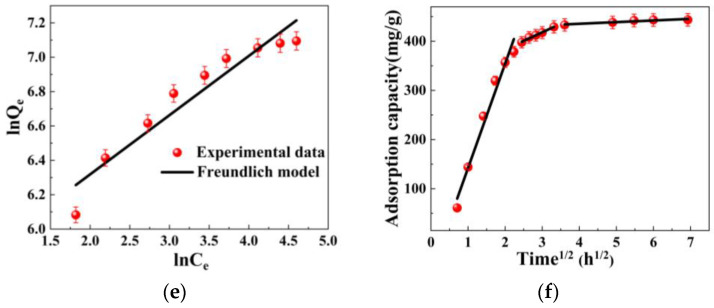
(**a**) Pseudo-first-order model; (**b**) Pseudo-second-order model; (**c**) Total adsorption isotherms curves; (**d**) Langmuir and (**e**) Freundlich adsorption isotherm curves; (**f**) Intraparticle diffusion model.

Linear plots between time t and log(Q_e_ − Q_t_)/t/Q_e_ are shown in Figure 5a,b and the corresponding rate constants and correlation coefficients (R^2^) are listed in Table 1. Clearly, the pseudo-second-order model showed a higher correlation coefficient (R^2^) than that of the pseudo-first-order model and Q_e_ deduced by the pseudo-second-order model was in good agreement with the experimental value (440 mg/g). These observations indicated that the MB adsorption processes over the SA/MX-0.74 NMs followed the pseudo-second-order kinetic model and were mainly affected by the chemical adsorption mechanism including ion exchange and/or shared electrons [69].

### 5.2. Adsorption Isotherm

The adsorption isotherm models of Langmuir and Freundlich were invoked to gain more insights into the relationship between initial MB concentrations and the adsorption effects. The Langmuir isotherm model assumes monolayer adsorption, wherein the adsorbent surface has the same active site and adsorption energy and there is no interaction between adsorbate molecules [70]. In contrast, the Freundlich isotherm model is used to illustrate multilayer adsorption occurring at heterogeneous interfaces [71]. The above isotherm models can be expressed as the following equations:

Freundlich isotherm:

(6) $${\mathrm{logQ}}_{e}{=\mathrm{logK}}_{F}+\frac{1}{n}{\mathrm{logQ}}_{e},$$

Langmuir isotherm:

(7) $$\frac{C_{e}}{Q_{e}}=\frac{1}{K_{L}Q_{m}}+\frac{C_{e}}{Q_{m}},$$

where Q_m_ (mg/g) is the maximum adsorption capacity; K_F_ [(mg/g)/(mg/L)^1/n^] and n are the Freundlich constant related to the adsorption capacity and adsorption intensity, respectively; and K_L_ (L/mg) is the Langmuir constant related to the maximum removal of energy.

Figure 5c–e displays the Langmuir and Freundlich isotherm models for MB adsorption over the SA/MX-0.74 NMs at 298 K and the corresponding fitted equilibrium data and constants with regression coefficients (R^2^) are presented in Table 2. It can be seen from Figure 5c, d that the adsorption processes obeyed the Langmuir model very well: the maximum adsorption capacity of the SA/MX-0.74 NMs for MB reaches 1371 mg/g, as the regression coefficient (R^2^) was higher than 0.99 (see Table 2). In contrast, the Freundlich model with a regression coefficient (R^2^) of 0.926 did not match the adsorption processes presented here well (see Figure 5e and Table 2). Indeed, the Langmuir model featuring monolayer adsorption was in accord with the chemical adsorption mechanism of the SA/MX-0.74 NMs, as indicated by the analyses of adsorption kinetics.

### 5.3. Intraparticle Diffusion

In order to further study the control steps of the adsorption rate, the Weber–Morris kinetic model was used to analyze the intraparticle diffusion mechanism, which can be expressed as the following equation [72]:

(8) $$Q_{t}{=K}_{w}t^{1/2}+C,$$

where K_w_ (mg/g h^1/2^) is the intraparticle diffusion rate constant (Weber–Morris rate constant); and C (mg/g) is the intercept that responds to the boundary layer thickness.

Figure 5f displays the linear-fitted plot based on the Weber–Morris kinetic model for MB adsorption over the SA/MX-0.74 NMs and the deduced parameters are listed in Table 3. Similarly to the results reported for SA-based adsorbents [28,61], three main steps occurred in the adsorption processes presented here. Firstly, a rapid adsorption featuring a high K_w_ value was observed (Part I, see Table 3), due to the fact that a high number of unoccupied adsorption sites existed on the adsorbent; secondly, as the adsorption sites were occupied, the adsorption rate slowed down and a moderate Kw value was observed (Part II, see Table 3); and finally, the adsorption sites were almost completely occupied and the adsorption reached equilibrium state (Part III, see Table 3). It should be noted that the K_w_ values at Part I and II observed for the SA/MX-0.74 NMs were much higher than those of SA-based adsorbents [28,61], which suggests that the adsorbent presented here possesses a higher adsorption rate due to the unique electrospinning-derived three-dimensional fibrous nanostructure.

### 5.4. Adsorption Thermodynamics

**Figure 6:**
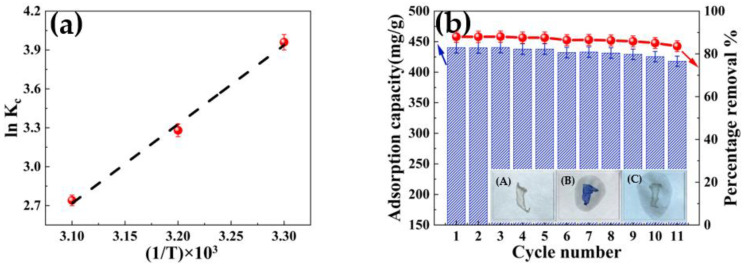
(**a**) Thermodynamic fitting curves of MB adsorption over SA/MX-0.74 NMs (T = 298, 308, and 318 K); (**b**) The reusability of SA/MX-0.74 NMs. (**A**) the photographs of fresh SA/MX-0.74 NMs; (**B**) MB-loading SA/MX-0.74 NMs after ten cycles; (**C**) the regenerated SA/MX-0.74 NMs after ten cycles.

In order to explore the spontaneity and feasibility of the adsorption processes, thermodynamic parameters, such as standard enthalpy (∆H^0^), standard entropy (∆S^0^), and standard Gibbs free energy (∆G^0^), were calculated [73] by using the following equations:

(9) $$K_{c}=\frac{Q_{e}}{C_{e}},$$

(10) $$\Delta G^{0}{=-\mathrm{RTlnK}}_{c},$$

(11) $${\mathrm{lnK}}_{c}=\frac{\Delta S^{0}}{R}-\frac{\Delta H^{0}}{\mathrm{RT}},$$

where K_c_, Q_e_, and C_e_ are the equilibrium constant, the equilibrium adsorption capacity of SA/MX-0.74 NMs (mg/g), and the equilibrium concentration of MB (mg/L), respectively; R is the gas constant (8.314 J/mol K); and and T is the adsorption temperature. The linear plot between lnK_c_ and 1/T × 10^3^ is shown in Figure 6a and the calculated thermodynamic parameters are listed in Table 4.

The negative values of ∆G^0^ at all the measured temperatures revealed the spontaneity of the MB adsorption processes presented here. Moreover, the value of ∆G^0^ increased with the increase in temperature, which implies that the higher temperature, the slower adsorption rate. This result was consistent with the fact that the adsorption processes presented here are exothermic, as confirmed by negative value of ∆H^0^ (−50.715 kJ/mol). The negative value of ∆S^0^ indicated that the randomness at the solid–solution interface decreased during the adsorption processes, possibly resulting from a more regular arrangement of MB molecules on the absorbents in comparison with the situation in aqueous solution.

### 5.5. Reusability of SA/MX-0.74 NMs

The reusability performance of adsorbents plays a crucial role in practical wastewater treatment. Following the optimum operational factors (T = 298 K, V = 30 mL, C_0_ = 50 mg/L, t = 24 h, pH = 7.0, dosage = 3 mg), the SA/MX-0.74 NMs were separated simply with tweezers, treated as described in Section 2.6, and then subjected to the subsequent adsorption. As can be seen from Figure 6b, after being reused ten times, the adsorption capacity and removal rate of the SA/MX-0.74 NMs decreased from 440 to 418 mg/g and from 88% to 84%, respectively. It should be noted that, even after being reused ten times, the SA/MX-0.74 NMs showed an MB adsorption capacity as high as 418 mg/g, which is higher than most of the NM materials reported previously (see Table 5). The fact that both the adsorption capacity and removal rate of the spent adsorbent that had been reused ten times could maintain 95% of the rates of the fresh adsorbent revealed that the sample of SA/MX-0.74 NMs was a rather robust adsorbent in treating MB-containing wastewater. Indeed, the regenerated SA/MX-0.74 NMs after ten cycles still retained almost identical morphology to that of the fresh one (see Figure 6b, inset).

### 5.6. Extended Experiments

**Figure 7:**
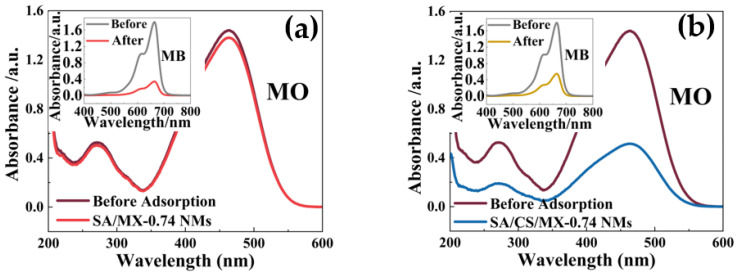
(**a**) UV-vis spectra of MO and MB before and after the adsorption over SA/MX-0.74 NMs; (**b**) UV-vis spectra of MO and MB before and after the adsorption over SA/CS/MX-0.74 NMs; (T = 298 K, V = 30 mL, C_0_ = 50 mg/L, t = 24 h, pH = 7.0, dosage = 3 mg).

As stated above, the sample of SA/MX-0.74 NMs, being a unique adsorbent with an electrospinning-derived three-dimensional fibrous nanostructure, demonstrated a high MB adsorption capacity and excellent reusability performance and the adsorption ability was associated with the electronegative surface of the MXene/SA composite fibers. In order to verify this electrostatic interaction, the SA/MX-0.74 NMs were subjected to the adsorption of anionic MO and the results are shown in Figure 7a. Clearly, except for physical adsorption, the SA/MX-0.74 NMs had no efficiency for adsorbing anionic MO molecules; indeed, this may be due to repulsive electrostatic interaction. Furthermore, to demonstrate the versatility of the electrospinning technique for fabricating highly efficient biomass adsorbents, an additional CS-containing sample, denoted as SA/CS/MX-0.74 NMs, was prepared by successively feeding SA/PVA/MXene and CS/PVA/MXene solution into the electrospinning apparatus. The amine moieties that exist in CS have been reported to have a high affinity with sulfonate moieties in anionic MO [80]. As shown in Figure 6b, the SA/CS/MX-0.74 NMs demonstrated a good adsorption performance for adsorbing anionic MO (325 mg/g, removal rate 65%) or cationic MB (380 mg/g, removal rate 76%). These results mean that, by designing sophisticated electrospinning processes, it is possible to fabricate amphoteric adsorbents with both electropositivity and electronegativity.

## 6. Conclusions

In summary, a series of novel SA/MXene (d-Ti_3_C_2_T_x_) composite nanofiber membranes were fabricated via electrospinning and successive Ca^2+^-mediated crosslinking processes. It was found that the content of MXene in the composite nanofiber membranes played a crucial role in the adsorption performance for adsorbing methylene blue. The adsorption performance of SA/MX-0.74 NMs with an MXene content of 0.74 wt.% showed an experimental adsorption capacity as high as 440 mg/g, and the maximum adsorption capacity was calculated as 1371 mg/g by a Langmuir isotherm model. Moreover, the adsorbent possessed excellent reusability and could be reused ten times without a significant loss of adsorption activity, as indicated by the fact that both the adsorption capacity and removal rate of the spent adsorbent could remain at 95% of those of the fresh one. It should be noted that amphoteric adsorbents with both electropositivity and electronegativity could be conveniently fabricated by designing sophisticated electrospinning processes, which demonstrates great potential for electrospinning techniques in fabricating highly efficient adsorbents associated with wastewater treatment.

## Figures and Tables

**Table 1:** Parameters of kinetic models for MB adsorption over SA/MX-0.74 NMs.

Q_e_ Exp (mg/g)	Pseudo-First-Order Model				Pseudo-Second-Order Model	
	K_1_ (h^−1^)	Q_e_ (mg/g)	R^2^	K_2_ (g/mg h)	Q_e_ (mg/g)	R^2^
440	0.199	231	0.961	1.595 × 10^−3^	441	0.998

**Table 2:** Parameters of isotherm models for MB adsorption over SA/MX-0.74 NMs.

Langmuir Isotherm Model			Freundlich Isotherm Model		
K_L_ (L/mg)	Q_m_ (mg/g)	R^2^	K_F_ [(mg/g)/(mg/L)^1/n^]	1/n	R^2^
0.083	1371	0.995	333.236	0.295	0.926

**Table 3:** Parameters of intraparticle diffusion model for MB adsorption over SA/MX-0.74 NMs.

	K_w_ (mg/g h^1/2^)	C (mg/g)	R^2^
Part I	209.89	−84.25	0.976
Part II	34.35	295.63	0.989
Part III	3.31	402.57	0.899

**Table 4:** Thermodynamic parameters for MB adsorption over SA/MX-0.74 NMs.

T (K)	∆G^0^ (kJ/mol)	∆H^0^ (kJ/mol)	∆S^0^ (J/mol K)
298	−10.596	−50.715	−134.629
308	−9.250		
318	−7.904		

**Table 5:** The equilibrium adsorption capacities of various nanofiber membranes for MB adsorption.

Adsorbents	Adsorption Capacity (mg/g)	References
PDA@DCA-COOH	61	[49]
Oxidized ECNFs	97	[74]
p(NIPAM-co-β-CD)/p(NIPAM-co-MAA)	132	[50]
CNFs	138	[51]
PVDF/PDA-30	173	[75]
CNFs@CNPs	222	[76]
PVA/Starch	381	[77]
PVA/PAA@PDA-15	446	[78]
Functionalized PES NFM	609	[79]
SA/MX-0.74 NMs	440	This study

## Data Availability

The data presented in this study are available on request from the corresponding author.

## Supplementary Materials

The following supporting information can be downloaded at: https://www.mdpi.com/article/10.3390/polym15092110/s1, Figure S1, photographs of (a) MXene powder; (b) SA Bs; (c) SA/MX-0.74 Bs; (d) SA NMs, and (e) SA/MX-0.74 NMs; Figure S2, SEM sectional image of SA/MX-0.74 NMs; Figure S3, XPS spectrum of SA/MX-0.74 NMs (Ti 2p); Figure S4, zeta potentials of SA/MX-0.74 NMs under different pH values. Video S1, the water contact angle measurement of SA/MX-0.74 NMs.
