# Supplementary material for: Electrospinning Novel Sodium Alginate/MXene Nanofiber Membranes for Effective Adsorption of Methylene Blue
Source: Polymers (Basel). 2023 Apr 28;15(9):2110. doi: 10.3390/polym15092110 (PMC10180889; doi:10.3390/polym15092110)
Supplement: Supplementary file 1 [file polymers-15-02110-s001.zip › polymers-2320914-supplementary.pdf]

Supporting Information

Electrospinning Novel Sodium Alginate/MXene Nanofiber Membranes for Effective Adsorption of Methylene Blue

Meng Li, Pingxiu Zhang, Qianfang Wang, Ningya Yu *, Xiaomin Zhang * and Shengpei Su

National and Local Joint Engineering Lab for New Petro-chemical Materials and Fine Utilization of Resources, Hunan Normal University, Changsha 410081, China; lm@smail.hunnu.edu.cn (M.L.); ZPX_serendipity@163.com (P.Z.); wqf199801@163.com (Q.W.); sushengpei@gmail.com (S.S.)

* Correspondence: yuningya@hotmail.com (N.Y.); zhangxm@hunnu.edu.cn (X.Z.); Tel.: +86-731-88872576 (N.Y.); Fax.: +86-731-88872531 (N.Y.)

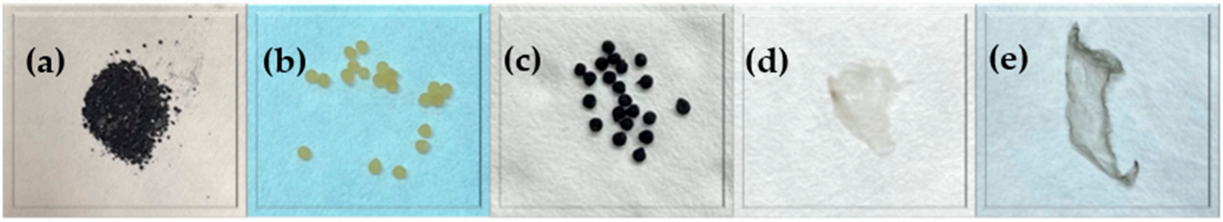

Figure S1. The photographs of (**a**) MXene powder, (**b**) SA Bs, (**c**) SA/MX-0.74 Bs, (**d**) SA NMs, and (**e**) SA/MX-0.74 NMs.

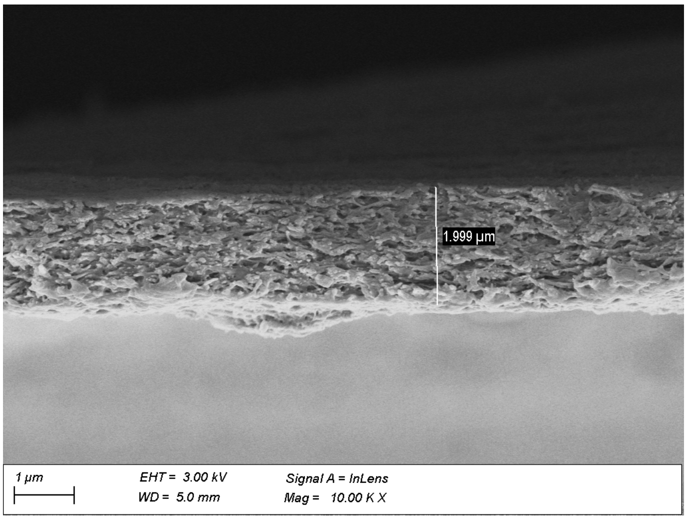

Figure S2. SEM sectional image of SA/MX-0.74 NMs.

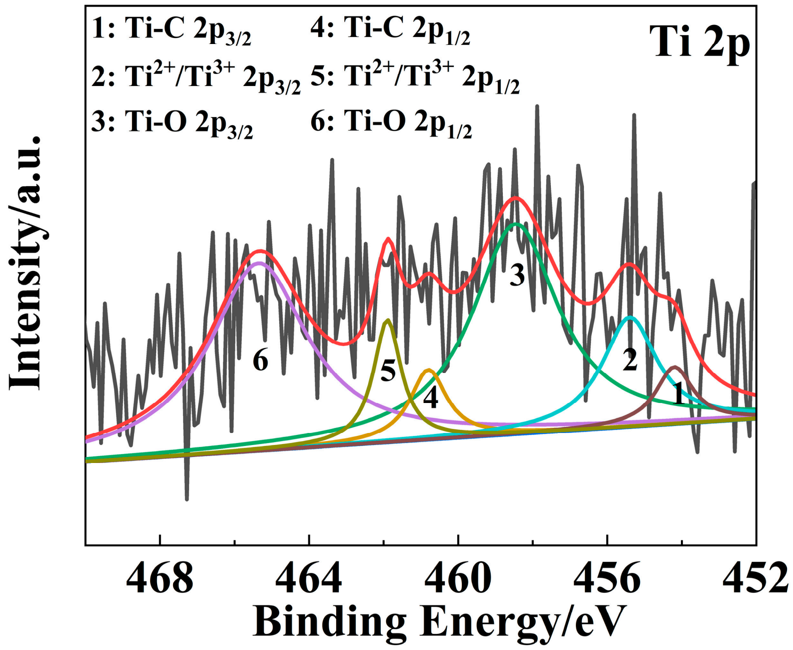

Figure S3. XPS spectrum of SA/MX-0.74 NMs (Ti 2p).

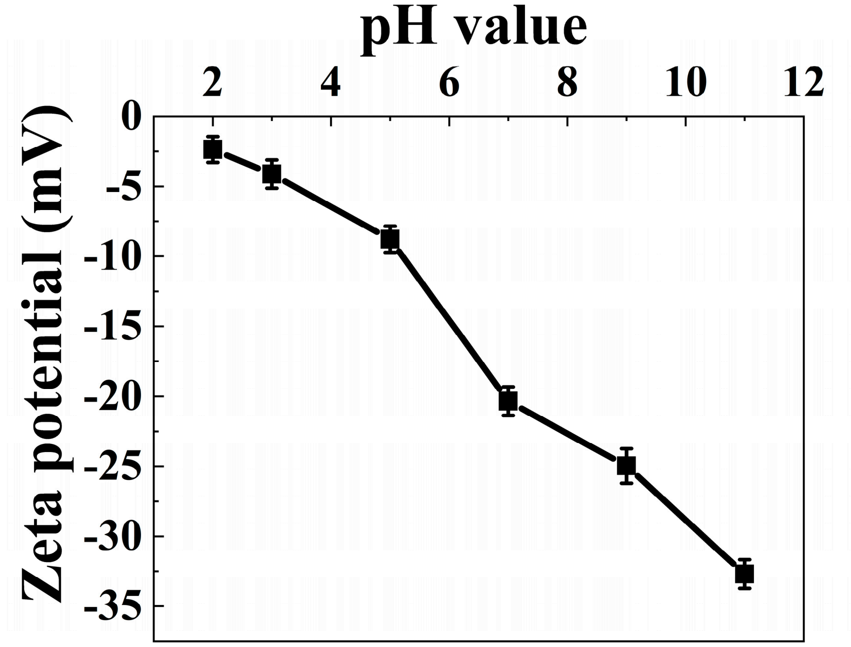

Figure S4. Zeta potentials of SA/MX-0.74 NMs under different pH values.

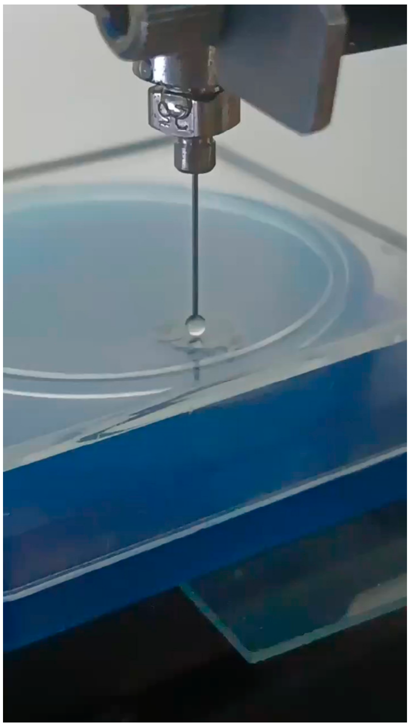

Video S1. The water contact angle measurement of SA/MX-0.74 NMs.
